# Accurate Measurement Methods of Frequency Eigenquantities in High-Speed Railway Seismic Wavefields and Applications to Distributed Acoustic Sensing Data

**DOI:** 10.3390/s26144387

**Published:** 2026-07-10

**Authors:** Yuhang An, Jihui Ma, Yunpeng Cai, Wenfa Yan

**Affiliations:** 1 Key Laboratory of Transport Industry of Big Data Application Technologies for Comprehensive Transport, Ministry of Transport, Beijing Jiaotong University, Beijing 100044, China; 21114020@bjtu.edu.cn (Y.A.); jhma@bjtu.edu.cn (J.M.); yanwenfa@bjtu.edu.cn (W.Y.); 2 Information Technology Center, Beijing Jiaotong University, Beijing 100044, China

**Keywords:** high-speed railway source, train frequency, bridge frequency, cepstral analysis, distributed acoustic sensing (DAS)

## Abstract

High-speed railways (HSRs) generate repeatable and spatially extended seismic wavefields, providing useful signals for distributed acoustic sensing (DAS)-based vibration analysis. This study develops an integrated measurement framework for two characteristic frequency eigenquantities in HSR-induced seismic wavefields: train frequency and bridge frequency. Building on established spectral-line, cepstral, and Doppler descriptions of HSR seismic wavefields, we systematize the relevant theoretical expressions, compare frequency-domain correlation and cepstral strategies for train-frequency estimation, and derive a velocity-independent bridge-frequency estimator from paired Doppler-shifted components. DAS data collected along viaduct sections of the Beijing–Guangzhou HSR are used to evaluate the framework across single trains, dense observation traces, and multiple train events. The results show that bridge frequency is more stable than train frequency, with lower measurement variance. The frequency-derived train speeds and carriage lengths fall within typical operating ranges of Chinese HSR trains, and the observed spatial periodicity in frequency measurements is consistent with bridge pier spacing. These findings support accurate frequency measurement and preliminary estimation of train speed, carriage length, and wave velocity from DAS records. Together, they clarify measurable frequency parameters of HSR seismic sources and establish a quantitative source-characterization basis for DAS-based railway vibration analysis and future multi-source monitoring studies.

## 1. Introduction

In recent years, railway transportation has become a central component of integrated transport systems. With the rapid expansion of high-speed railway (HSR) networks, the safe operation of bridges, tracks, and subgrades requires monitoring methods that are continuous, economical, and sensitive to small changes in vibration behavior [1]. For seismic and distributed fiber-optic monitoring, the key issue is therefore not only whether train-induced vibrations can be detected, but also whether the repeatable wavefield generated by HSR operation can be described by quantitative source parameters that remain comparable across sensors, trains, and observation periods.

Modern safety monitoring is also moving toward data-driven vibration-based paradigms that extract diagnostic information from operational vibration responses rather than relying only on visual inspection or sparse manual measurements. For example, Hacıefendioğlu et al. developed an unsupervised structural-health-monitoring framework based on vision-based vibration analysis and a dual-domain deep convolutional variational autoencoder [2]. Such studies illustrate a broader trend toward using vibration records as quantitative monitoring data; the present work follows this trend from a distributed fiber-optic perspective by focusing on recoverable frequency eigenquantities in HSR-induced seismic wavefields.

Previous studies have demonstrated that high-speed trains can act as repeatable mobile seismic sources for subsurface imaging and wavefield analysis [3,4,5,6]. In parallel, distributed acoustic sensing (DAS) has enabled existing telecommunication or trackside optical fibers to be used as dense vibration arrays for seismic observation and railway or traffic monitoring [7,8,9,10,11,12,13,14]. Recent DAS-based traffic and railway studies have further shown that vehicle or train events can be detected, tracked, classified, and used for velocity estimation or near-surface imaging [15,16], while broader DAS applications have demonstrated the value of fiber-optic arrays for seismic monitoring and near-surface studies [17,18,19,20]. These studies establish the feasibility of using traffic-induced DAS records, but most of them focus on event monitoring, target tracking, or imaging outcomes rather than on the accurate measurement of the source-frequency eigenquantities that control the HSR-induced seismic spectrum.

The need for such frequency measurements arises from the particular source mechanism of HSR-induced seismic waves. Conventional seismic sources can often be approximated as short-duration point sources, whereas an HSR train is a moving combined source that continuously excites the railway line. Two periodicities are especially important. First, the repeated loading of railcars, bogies, and axles generates equidistant spectral lines related to train speed and vehicle geometry [21,22,23,24,25]. Second, on viaduct sections, regularly spaced bridge piers are excited sequentially, producing bridge-frequency components that may be Doppler shifted before and after train passage [26,27]. Existing train-speed estimation methods provide useful operational parameters [23], spectral-line studies reveal the frequency spacing caused by train structure [24,25], and Doppler analyses explain the shifted bridge-frequency components [27]. However, these approaches do not fully solve the measurement problem addressed here: train-speed methods do not quantify the uncertainty of the underlying frequency eigenquantity, spectral-line and cepstral applications have not been systematically compared with correlation-based estimators for HSR DAS records, and previous Doppler studies did not directly provide a velocity-independent field estimator for the intrinsic bridge frequency.

The current literature can therefore be grouped into three method families. First, DAS-based railway and traffic-monitoring studies make effective use of dense fiber-optic arrays for traffic-flow detection, vehicle classification, object tracking, velocity estimation, and near-surface imaging [10,11,15,16], but they generally treat train-induced vibrations as events or imaging sources rather than as repeatable sources with measurable frequency eigenquantities. Second, studies on train vibration spectra and train-speed estimation identify equidistant spectral lines and relate their spacing to vehicle geometry and operating speed [23,24,25], yet they mainly focus on train-frequency or speed retrieval and have not systematically compared correlation-based and cepstrum-based estimators under identical DAS observation conditions. Third, theoretical and observational studies of viaduct–ground interaction, train-induced seismic interferometry, HSR seismic imaging, and Doppler effects have clarified bridge-pier excitation, recoverable wavefield components, and shifted bridge-frequency components [21,22,26,27,28,29,30,31,32,33], but they do not provide a field workflow that combines paired shifted components to recover a velocity-independent intrinsic bridge frequency. This comparison defines the scope of the present paper: not to replace these existing methods, but to connect them into a measurement-oriented framework for train frequency and bridge frequency with trace-level uncertainty assessment.

Based on the above comparison, the present study focuses on the methodological link between HSR source physics and DAS-based field measurement. Specifically, we (1) summarize the physical expressions of train frequency and bridge frequency in a unified notation, (2) compare frequency-domain correlation and cepstral analysis for quantitative train-frequency estimation, clarifying that cepstral analysis is used here for direct measurement of spectral spacing rather than only for train-formation identification, and (3) formulate and validate a velocity-independent bridge-frequency estimator using paired Doppler-shifted spectral peaks. The distinction from existing spectral-line, cepstral, Doppler-based, and DAS monitoring studies is therefore not the discovery of an isolated new spectral phenomenon, but the construction of a field-oriented measurement workflow that links these known physical effects to recoverable frequency eigenquantities and evaluates their uncertainty with dense DAS observations. By applying this framework to DAS data recorded along the Beijing–Guangzhou HSR, we evaluate measurement uncertainty across dense sensing traces and multiple train passages, thereby addressing a gap between existing train-speed, spectral-line, Doppler, and DAS monitoring studies.

## 2. Frequency Characteristics in HSR-Induced Seismic Wavefields

This study focuses on the seismic wavefield generated by high-speed trains running over bridge sections. Theoretical expressions for such wavefields are presented below. The bridge piers, assumed to be uniformly spaced with an interval of D, are treated as point sources. For simplification, we consider a scalar wavefield and assume a homogeneous medium with constant wave velocity c. This wave velocity could represent the propagation speed of P-waves, S-waves, or surface waves in subsurface media, with its specific nature determined by the energy proportion of each wave type in the actual recorded signals. Under these assumptions, the HSR-induced wavefield generated by multiple bridge piers can be expressed as follows [6]:

(1) $$u\left(x,t\right)=\sum_{j}{ց}_{j}\left(x,t;\xi_{j},\tau \right)*s_{j}\left(t\right)$$

In this expression, the recorded wavefield is represented as the convolution of the Green’s function ${ց}_{j}$ between source location $\xi_{j}$ and receiver location x, and the source time function $s_{j}\left(t\right)$ of the *j*-th pier. The total wavefield results from the superposition of contributions from all piers. We assume that each bridge pier is excited independently, and the mutual scattering between piers can be neglected since the wavelengths of interest (hundred-meter scale) are significantly larger than the pier dimensions (meter scale).

Assuming the train moves at a constant velocity v, the excitation of the j-th pier is delayed relative to the initial pier by $\frac{jD}{v}$, leading to the following expression for its source time function:

(2) $$s_{j}\left(t\right)=s_{0}\left(t-\frac{jD}{v}\right)$$

Here, $s_{0}$ is the source time function of the reference (initial) pier. This formulation describes the sequential excitation of piers as the train moves forward.

Substituting Equation (2) into Equation (1) and transforming to the frequency domain, the time delay $t-\frac{jD}{v}$ becomes a phase shift $e^{-i\omega jD/v}$. Meanwhile, assuming a homogeneous medium, Green’s function (neglecting amplitude variations) simplifies to a phase term $e^{-i\omega r_{j}/c}$, where $r_{j}$ is the distance from the j-th pier to the receiver, and ω is the angular frequency, defined as ω=2πf. The frequency-domain wavefield is then:

(3) $$U\left(x,\omega \right)=\sum_{j}e^{-i\omega r_{j}/c}S_{0}\left(f\right)e^{-i\omega jD/v}=S_{0}\left(\omega \right)\sum_{j}e^{-i\omega \left(jD/v+r_{j}/c\right)}$$

The frequency-domain wavefield in Equation (3) consists of two key components: the source spectrum $S_{0}\left(\omega \right)$ of an individual bridge pier and the phase summation term $\sum_{j}e^{-i\omega \left(jD/v+r_{j}/c\right)}$ resulting from the collective excitation of multiple piers. These components respectively encode two primary frequency characteristics of HSR-induced sources—train frequency, arising from the quasi-periodic excitation of railcars, and bridge frequency, caused by the regular spatial distribution of bridge piers. In the following sections, we provide a detailed analysis of the physical significance and extraction methodology of these frequency features. It should be emphasized that the assumption of a homogeneous medium was adopted in the above derivation for simplicity. Under more complex geological conditions, the measurements of frequency-dependent parameters can be influenced by local heterogeneities, and this aspect will be specifically addressed in the subsequent discussions of the two frequency-related characteristic quantities.

The homogeneous-medium assumption is therefore an intentional first-order approximation, following previous theoretical treatments of HSR seismic wavefields [6,27], rather than a complete description of the actual site. The HSR seismic source is already a distributed and time-dependent source involving repeated excitation by multiple bridge piers, carriages, bogies, and wheelsets. If full lateral heterogeneity, scattering, mode conversion, and local coupling variations were introduced at the same stage, the model would become difficult to analyze quantitatively and the source-controlled periodicity would be obscured. We therefore use a scalar effective-velocity model to derive the leading-order frequency relationships. In a non-uniform medium, local velocity variations and coupling differences may perturb phase and amplitude, broaden spectral peaks, and generate trace-dependent outliers; however, the characteristic frequency associated with regularly spaced bridge piers remains primarily controlled by the excitation interval D/v. The field analysis below therefore treats the idealized derivation as a measurement guide and evaluates deviations empirically through multi-trace uncertainty, harmonic consistency, and outlier analysis.

### 2.1. Carriage Repetition and “Train Frequency”

The “train frequency” primarily arises from the periodic excitation induced by each carriage as the train moves, leading to an evenly spaced discrete spectral structure in the frequency domain [23,24,25].

Assuming that the train consists of N carriages, the source time function can be expressed as follows:

(4) $$S_{0}\left(\omega \right)=S_{c}\left(\omega \right)\sum_{k=1}^{N}e^{-i\omega kL/v}$$

Here, L denotes the length of a single carriage, and $S_{c}\left(\omega \right)$ represents the source time function generated as one carriage passes by. More specifically, the source function of a single carriage can be further decomposed into the superposition of signals from its four sets of wheels:

(5) $$S_{c}\left(\omega \right)=S_{w}\left(\omega \right)\sum_{k=1}^{4}e^{-i\omega d_{k}/v}$$

In Chinese HSR systems, each carriage typically has four sets of wheels, with their relative positions denoted by $d_{k}$. The term $S_{w}\left(\omega \right)$ represents the source time function generated by a single wheelset, which can be reasonably approximated as a broadband source function.

**Figure 1:**
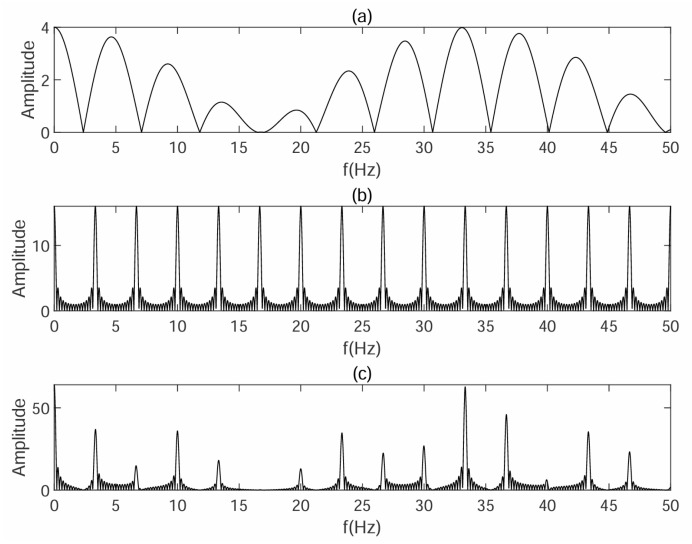
Frequency spectrum of the HSR source time function. (**a**) Modulation by wheelsets; (**b**) Modulation by carriages; (**c**) Combined modulation of wheelsets and carriages.

Figure 1 illustrates the frequency-domain characteristics of the source function under three scenarios: (a) superposition of four wheelsets, (b) superposition of 16 carriages, and (c) combined modulation from both wheelsets and carriages. The results show that the wheelsets introduce two distinct modulation cycles in the spectrum, while the carriage-level repetition produces sharp, evenly spaced spectral peaks—characteristic of a discrete line spectrum. When both effects are present, the spectral shape remains predominantly governed by the carriage modulation.

Accordingly, the train frequency describes the temporal repetition rate of carriages and can be expressed as: $f_{c}=v/L$. Using typical parameters for Chinese high-speed trains, with an operating speed of approximately 300 km/h (equivalent to 83.3 m/s) and a carriage length of about 25 m, the characteristic train frequency is approximately 3.3 Hz. Since this expression does not involve the wave velocity, this frequency-dependent parameter is not affected by the complexity of the subsurface medium.

### 2.2. Superposition of Multiple Bridge Piers and the “Bridge Frequency”

The first characteristic frequency in the HSR-induced seismic wavefield—the train frequency—is determined by the source time function $S_{0}\left(\omega \right)$, whereas the second, known as the bridge frequency, is governed by the phase interference term $\sum_{j}e^{-i\omega \left(jD/v+r_{j}/c\right)}$, resulting from the sequential excitation of bridge piers. Unlike the source function, this term depends not only on the train speed v and the bridge pier spacing D, but also on the seismic wave velocity c of the medium and the location of the receiver.

To facilitate analysis, we make a simplification: under typical conditions, the seismic wave velocity c is much greater than the train speed v. As a result, the contribution from the second term $r_{j}/c$ in the phase expression can be considered negligible compared to jD/v. Therefore, the dominant frequency resulting from the periodic excitation of bridge piers can be approximated as: $f_{p}=v/D$.

For instance, in Chinese high-speed railway systems, the average spacing between bridge piers is approximately 32 m, and combined with the aforementioned typical train speed yields a typical bridge frequency of 2.5 Hz.

When the seismic wave velocity c (specifically near-surface S-wave and surface wave velocities) is not significantly greater than the train speed v, the bridge frequency becomes sensitive to the location of the observation point relative to the moving train. In this study, we focus on receivers positioned along HSR bridge segments. According to the analysis of Jiang et al. [27], the phase summation terms for observation points located in front of (train approaching) and behind (train receding) the train can be expressed as follows:

(6) $$\sum_{j}e^{-i\omega jD\left(1/v-1/c\right)}\;(\text{train}\;\text{approaching})$$

(7) $$\sum_{j}e^{-i\omega jD\left(1/v+1/c\right)}\;(\text{train}\;\text{receding})$$

These correspond to the following shifted frequencies:

(8) $$f_{1}=\frac{cv}{D\left(c-v\right)}\;(\text{train}\;\text{approaching})$$

(9) $$f_{2}=\frac{cv}{D\left(c+v\right)}\;(\text{train}\;\text{receding})$$

**Figure 2:**
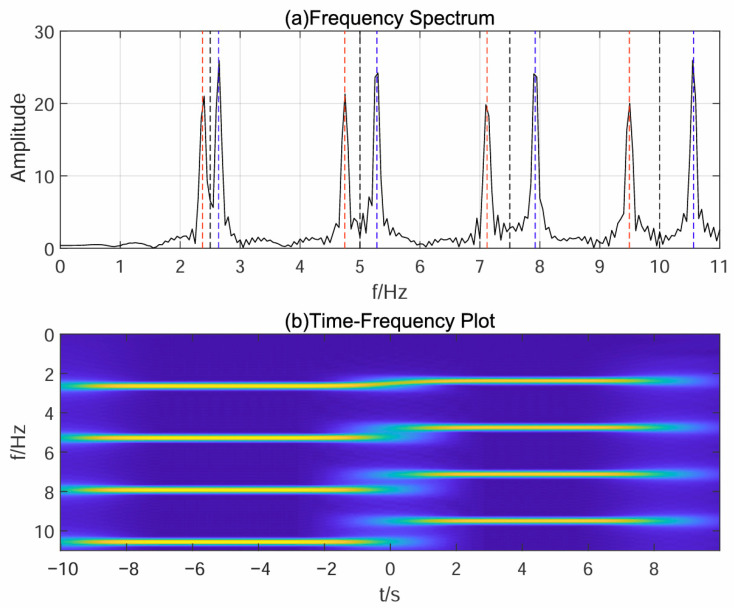
Doppler-induced frequency shift under bridge-frequency modulation. (**a**) Amplitude spectrum; (**b**) Time–frequency representation. Black dashed lines indicate the bridge frequency and its harmonics, while blue and red dashed lines correspond to the blue-shifted (before train arrival) and red-shifted (after train departure) frequencies, respectively. The yellow regions in (**b**) indicate higher spectral amplitudes.

Such frequency shifts are manifestations of the classical Doppler effect, analogous to the Doppler shifts observed in electromagnetic or acoustic waves [27]. Figure 2 illustrates the shift in the bridge frequency under different observational scenarios. The physical basis of the approaching and receding frequency components follows the analysis of Jiang et al. [27]. In the present study, we use this established Doppler framework as the starting point for a measurement problem: recovering the intrinsic bridge frequency from the two shifted components without requiring prior knowledge of the wave velocity. The Doppler effect described in the above derivation corresponds to the case where the source is moving while the receiver remains stationary. Under this condition, if medium heterogeneity is taken into account, the wave velocity appearing in the corresponding expression should be interpreted as the wave velocity at the source location (i.e., at the position of the moving train).

## 3. Method for Measuring the “Train Frequency”

Measuring the train frequency is essentially the task of estimating the spacing of an equidistant discrete spectrum generated by the periodic passage of train carriages. In this section, we compare two measurement routes rather than presenting cepstral analysis as a new signal-processing transform. The frequency-domain correlation method estimates the spacing indirectly by searching for the theoretical spectrum that best matches the observed spectrum, whereas the cepstral method estimates the same spectral periodicity directly from the peak in the quefrency domain. This comparison is useful because the two methods have different assumptions and practical trade-offs in search efficiency, parameter dependence, and frequency resolution.

### 3.1. Spectral Correlation Coefficient Search Methods

Frequency-domain correlation is one of the most commonly used approaches in previous studies for estimating train speed and its associated frequency characteristics. The method is conceptually straightforward: it involves computing the cross-correlation between the observed seismic spectrum of the high-speed train and a theoretical reference spectrum generated from assumed values of carriage length and train velocity. The parameter set corresponding to the maximum correlation coefficient is then taken as the estimated value of the train frequency (or speed).

**Figure 3:**
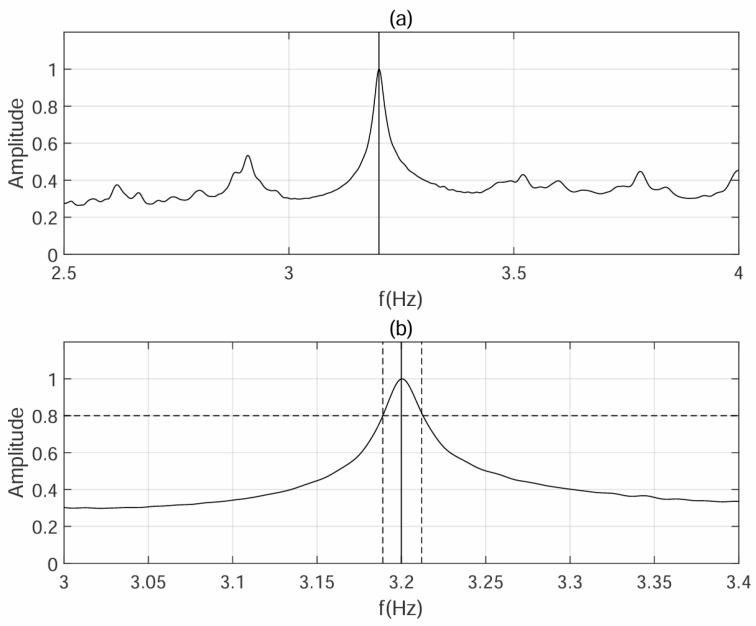
Train-frequency estimation using frequency-domain correlation. (**a**) Correlation coefficients over the full frequency range; (**b**) Zoomed-in view of (**a**). The black solid line indicates the model-defined train frequency of 3.2 Hz, while the black dashed lines mark the threshold at 80% of the maximum correlation coefficient and the corresponding frequency range.

We follow previous studies [32] and compute the normalized correlation coefficient between the observed spectrum and a theoretical spectrum constructed with a prescribed “train frequency”:

(10) $$corr\left(f_{c}\right)=\frac{\int_{\omega_{1}}^{\omega_{2}}\left|S_{1}\left(f_{c},\omega \right)\right|\left|Y\left(\omega \right)\right|d\omega }{\sqrt{\int_{\omega_{1}}^{\omega_{2}}{\left|S_{1}\left(f_{c},\omega \right)\right|}^{2}d\omega }\sqrt{\int_{\omega_{1}}^{\omega_{2}}{\left|Y\left(\omega \right)\right|}^{2}d\omega }}$$

where $S_{1}\left(f_{c},\omega \right)=\sum_{k=1}^{N}e^{-i\omega k/f_{c}}$ denotes the theoretical spectrum shown in Figure 3b, and $Y\left(\omega \right)$ represents the observed spectrum.

To evaluate the measurement accuracy and error characteristics of the correlation-based method, we conducted a series of simulations using synthetic data. In the first scenario, we set the train speed to 80 m/s and the carriage length to 25.0 m, resulting in a theoretical train frequency of 3.2 Hz. We computed the spectrum under the joint modulation of carriages and wheelsets in this scenario (i.e., the algorithm shown in Figure 1c) and used it as the reference spectrum for the observation.

Frequency-domain correlation was then applied using a search step of 0.001 Hz.

The correlation coefficients between the simulated spectrum at different frequencies and the reference spectrum were calculated and normalized across frequencies. The results are shown in Figure 3. As shown, the maximum correlation coefficient occurs precisely at 3.2 Hz, matching the theoretical value. To quantify measurement uncertainty, we defined a threshold at 80% of the peak correlation coefficient and computed the frequency range satisfying this condition. The resulting standard deviation of the estimated frequency is approximately 0.010 Hz, indicating high measurement accuracy.

### 3.2. Cepstral Method

Frequency-domain correlation is effective and, as shown below, can provide high-resolution train-frequency estimates when an appropriate reference spectrum and search interval are specified. Its measurement logic, however, is template based: a series of candidate train frequencies or train speeds must be tested, and the result depends on the assumed source model, search range, and frequency step. Cepstral analysis provides a complementary route because it transforms the regular spacing of spectral lines into a peak in the quefrency domain and therefore reads the spacing more directly.

Given the prominent evenly spaced spectral lines in HSR-induced wavefields, cepstral analysis provides a direct way to estimate the spacing of periodic structures in the spectrum. This use is consistent with standard cepstral analysis: just as a Fourier transform is a direct tool for identifying temporal periodicity in a time-domain signal, the cepstrum is a direct tool for identifying periodicity in the frequency domain. We note that cepstral attributes have already been used in HSR signals to identify carriage length, carriage number, and train formation patterns [23]. The purpose here is different: we use the cepstral peak as a quantitative estimator of the train-frequency eigenquantity fc, evaluate its uncertainty, and compare its performance with the frequency-domain correlation method under the same synthetic and DAS data conditions. Thus, the contribution is not the introduction of a new cepstral algorithm, but the formulation and assessment of a direct train-frequency measurement strategy for HSR-induced seismic spectra.

The cepstrum is computed by taking the logarithm of the signal’s spectrum and then applying a Fourier transform (not an inverse transform). The resulting cepstral domain has a time-like dimension, often referred to as quefrency. Periodic structures in the frequency domain (such as discrete line spectrum) manifest as distinct peaks in the cepstrum.

By identifying the peak position in the cepstrum, one can estimate the temporal spacing τ between excitations from adjacent carriages. The corresponding train frequency is then calculated as follows:

(11) $$f_{c}=1/\tau$$

where τ is the quefrency associated with the cepstral peak, representing the carriage excitation interval.

**Figure 4:**
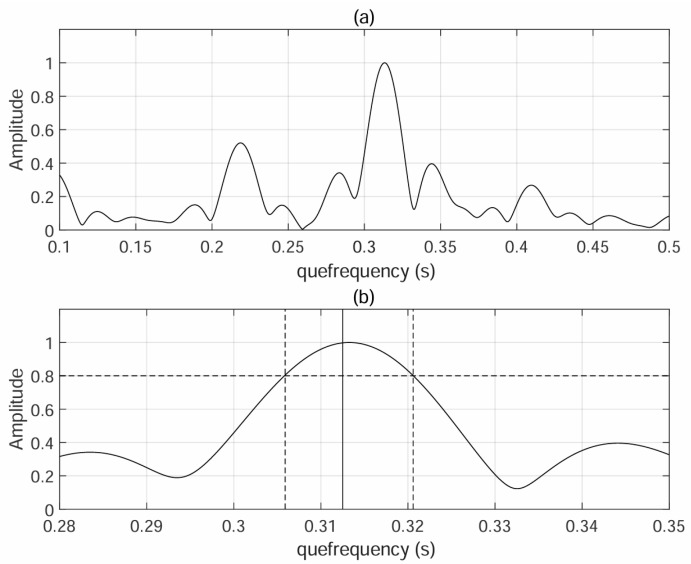
Train-frequency estimation using cepstral analysis. (**a**) Cepstrum; (**b**) Enlarged view of (**a**). The black solid line marks the quefrency corresponding to the model-defined train frequency (0.3125 s), while the black dashed lines indicate the threshold at 80% of the peak amplitude and the associated quefrency interval.

For the synthetic spectrum generated with a train speed of 80 m/s and a carriage length of 25.0 m, the corresponding cepstrum is shown in Figure 4. The peak position in the cepstrum aligns precisely with the expected quefrency of 0.3125 s, which corresponds to the model-defined train frequency, confirming the accuracy of the method.

Using the same threshold—80% of the peak amplitude—to assess measurement uncertainty, the resulting quefrency range is 0.3059–0.3206 s. This translates to a frequency range of approximately 3.12–3.27 Hz. The computed standard deviation of the estimated frequency is slightly larger than that obtained using the frequency-domain correlation method. This indicates that while cepstral analysis has the advantage of requiring no prior parameter assumptions, it may offer slightly lower frequency resolution compared to correlation-based approaches.

The above comparison should be interpreted as a relative error analysis rather than a universal ranking of the two methods. The 80% peak-width criterion is used as a common diagnostic of peak sharpness under identical synthetic-data conditions; it is not intended to represent the true statistical standard deviation of field measurements. For the frequency-domain correlation method, the main error sources include mismatch between the observed source spectrum and the assumed reference spectrum, uncertainty in carriage or wheelset geometry, finite search step, spectral leakage caused by the finite time window, and noise or local coupling changes that alter the relative amplitudes of spectral lines. Therefore, this method can provide high frequency resolution when the reference model is appropriate, but it is model dependent. For the cepstral method, the main error sources include the finite frequency bandwidth of the spectrum, smoothing and leakage of the log-spectrum, interference from broad spectral trends, weak or missing harmonic lines, and the resolution of the quefrency-domain peak. Therefore, cepstral analysis is more direct and requires fewer prior train parameters, but its peak can be broader or shifted when the equidistant spectral lines are weak. The two approaches are thus complementary: frequency-domain correlation is preferable when a reliable reference spectrum is available, whereas cepstral analysis is useful as a parameter-light estimator and as an independent check on the spectral-line spacing.

## 4. Measurement of Bridge Frequency and Its Applications

### 4.1. Method for Measuring the Bridge Frequency

As discussed in Section 2.2, the bridge frequency is defined as follows:

(12) $$f_{p}=v/D$$

This characteristic frequency depends only on the train speed v and the bridge pier spacing D, and is independent of the seismic wave velocity c.

Although the Doppler-shifted bridge-frequency components before and after train passage have been described in previous studies [27], the intrinsic bridge frequency fp is not directly observed in field spectra. In this study, we combine the two observed components as a measurement step so that the dependence on wave velocity is eliminated. This leads to the following velocity-independent estimator:

(13) $$\frac{2}{f_{p}}=\frac{1}{f_{2}}+\frac{1}{f_{1}}$$

Equation (13) is the explicit bridge-frequency estimator used in this study. The derivation is algebraically simple, but previous Doppler analyses did not directly use this reciprocal combination of the approaching and receding frequency components to recover the intrinsic bridge frequency from field observations. Thus, the novelty of the bridge-frequency part of this paper lies in the velocity-independent formula, its implementation with dense DAS records by pairing pre-arrival and post-departure spectral peaks, and its validation using single-trace, multi-trace, and multi-train field measurements.

For the characteristic frequencies corresponding to the time windows before and after train passage, the paired peaks should be selected at the same harmonic order. In field DAS spectra, the fundamental bridge-frequency component can be weak because the train is far from the observation point in these windows and because low-frequency energy may be mixed with other wavefield components. We therefore examine the fundamental component and its harmonics, then select the paired peaks with higher signal-to-noise ratios and consistent harmonic order. In the data analyzed below, the second harmonic near 5 Hz and the fourth harmonic near 10 Hz provide more stable peak pairs than the fundamental component, and the harmonic order is accounted for when converting the measured shifted peaks to the intrinsic bridge frequency fp.

This harmonic-selection strategy connects the theoretical velocity-independent expression with practical DAS implementation: the pre-arrival and post-departure peaks are first paired within the same harmonic band, and Equation (13) is then applied to obtain a robust estimate of the bridge frequency.

### 4.2. Estimation of Train Speed and Wave Velocity

Once the bridge frequency is measured, the train speed can be easily estimated using the known pier spacing:

(14) $$v=f_{p}\cdot D$$

To further estimate the wave velocity, the following Doppler-based formulation can be used with the measured f1 and f2 [27]:

(15) $$c=\frac{2D}{\frac{1}{f_{2}}-\frac{1}{f_{1}}}$$

The wave velocity estimated in this way should be interpreted as an apparent propagation velocity associated with the Doppler-shifted bridge-frequency components. This technical route is not an ad hoc assumption introduced in the present study; it follows the established Doppler-effect formulation for HSR-induced seismic wavefields [27]. Equation (15) is used only after the approaching and receding peaks are identified from paired spectral components with the same harmonic order. The main error sources are peak-picking uncertainty, phase mixing among P-waves, S-waves, surface waves, and bridge-guided waves, and local variations in DAS cable–ground coupling. In the present workflow, these errors are mitigated by selecting higher-SNR same-harmonic peak pairs, constraining the peak search to expected frequency bands, checking the spectra against background noise, retaining outliers in the multi-trace analysis, and quantifying trace-to-trace and train-to-train scatter. These steps do not eliminate all ambiguity in wave-type identification; so, the result should not be regarded as an independently verified true site velocity without additional calibration. Instead, it provides a passive, HSR-source-based apparent velocity estimate that can be used as a consistency check and as a basis for future near-surface velocity studies along railway corridors.

## 5. Application of the Measurement Method to DAS Data

### 5.1. Experimental Setup

**Figure 5:**
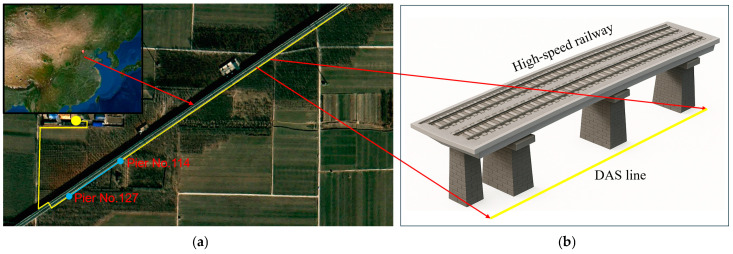
Study area and DAS deployment schematic for the elevated high-speed railway. (**a**) Satellite photograph of the data acquisition area; (**b**) Layout of the high-speed rail and DAS line.

From November to December 2024, a continuous monitoring campaign lasting approximately one month was conducted. Figure 5 presents the study site and the schematic of the DAS deployment. The monitoring location is situated on an elevated section of the Beijing–Guangzhou High-Speed Railway near Liutuo Village, Wangdu County, Baoding City, Hebei Province.

In the satellite image (Figure 5a), the yellow line indicates approximately 2.5 km of fiber-optic cable buried in a shallow trench alongside the viaduct; approximately 2 km of the cable was installed parallel to the bridge span. The yellow circle in Figure 5a denotes the location of the DAS interrogator. The viaduct cross-section (Figure 5b) consists of a concrete deck supporting two parallel steel rails and evenly spaced bridge piers; the yellow line denotes the route of the distributed acoustic sensing (DAS) cable. During installation, a narrow trench approximately 0.2 m deep was excavated along the viaduct, and two types of fiber were buried: (1) GYXTW-type 4-core armored outdoor cable and (2) standard indoor drop cable.

The DAS system was a HiFi-DAS unit (Puniu Technology (Shanghai, China)) configured with a 500 Hz sampling rate, 0.8 m trace spacing, and a 1.6 m gauge length, with a response frequency range of 0.001–100 kHz. The system recorded single-component axial strain rate along the fiber. This deployment provided high-resolution recordings of the seismic wavefield generated by passing high-speed trains and a robust dataset for extracting and analyzing train and bridge spectral characteristics.

**Figure 6:**
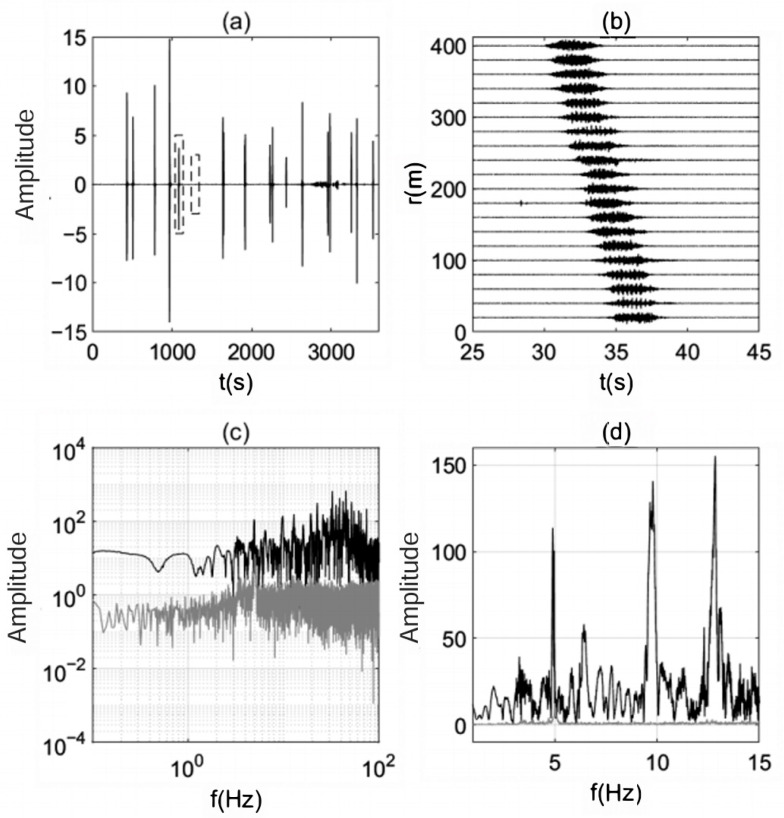
One-hour DAS recording of high-speed train seismic signals. (**a**) One-hour record from trace 1 showing ~20 train passages, The left and right dashed boxes indicate a representative train event and a background-noise segment, respectively; (**b**) zoom-in of the left dashed box in (**a**) from 20 traces (20 m spacing); (**c**) logarithmic amplitude spectrum of train signals (black lines) and background noise (gray lines); (**d**) linear amplitude spectrum highlighting peaks near 5 Hz and 10 Hz.

For method validation, we selected a one-hour dataset recorded on 23 November 2024 from 07:30 to 08:30 UTC, using channels 501–1000, which correspond to the section between bridge piers No. 114 and No. 127 (highlighted in blue in Figure 5a). The dataset was used to analyze vibrations induced by passing high-speed trains and the corresponding background noise. Data from trace 1 are displayed in Figure 6a, where multiple distinct vertical streaks correspond to seismic responses generated by high-speed trains. During this period, approximately 20 trains passed through the observation area. Figure 6b zooms in on one train event (outlined by the left dashed box in Figure 6a) and shows signals from 20 traces spaced at 20 m intervals, revealing the motion of the train from distal to proximal locations. To compare train-induced signals with background noise, Figure 6c,d present the logarithmic and linear amplitude spectra of the two boxed signals in Figure 6a, respectively. The train-induced signals are significantly stronger than the background noise and show distinct peaks near 5 Hz and 10 Hz.

### 5.2. Measurement Results from a Single Train at a Single Observation Point

**Figure 7:**
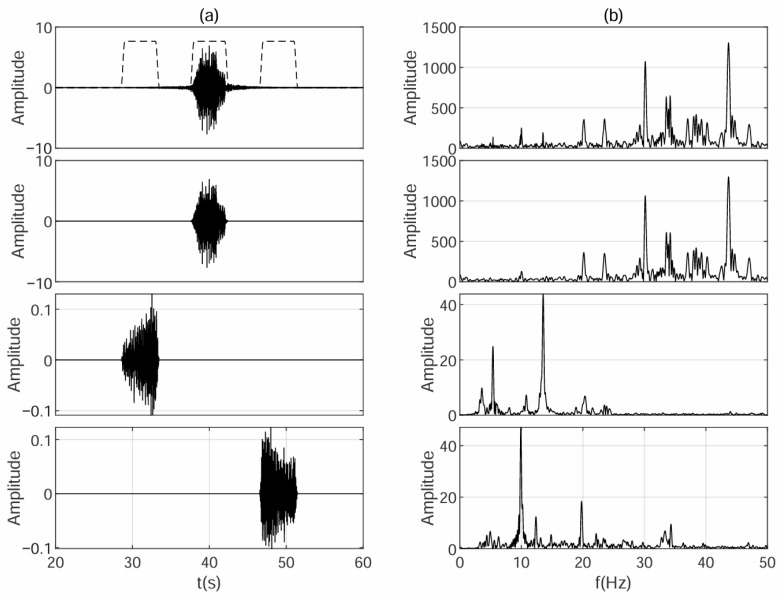
High-speed train signal and spectral analysis used for extracting the train and bridge frequencies. (**a**) Time-domain signals over a 40-s window: the first row shows the full waveform, and the second to fourth rows show 5-s segments during train passage, before arrival, and after departure, respectively; (**b**) amplitude spectra corresponding to the three segments, used for estimating train frequency and bridge frequency.

From the DAS array, we selected an observation point near the southern end and extracted a 40-s data segment centered on the passage of a single high-speed train—20 s before and 20 s after the train reached the site. This waveform is shown in Figure 7a. As the train approaches the observation point, the recorded seismic amplitudes increase significantly, yielding a high signal-to-noise ratio. In contrast, signals recorded before and after the train passage decay rapidly in amplitude.

Three 5-s time windows are indicated with dashed lines in Figure 7a, corresponding to the pre-arrival, passage, and post-departure stages of the high-speed train, respectively. The windows were selected using a fixed and reproducible procedure. First, we identified the train-center time t0 as the time for which the waveform envelope was most symmetric around the train passage. The passage window was then defined as the 5-s interval from $t_{0}-2.5$ s to $t_{0}+2.5$ s. The centers of the pre-arrival and post-departure windows were set to $t_{0}-9$ s and $t_{0}+9$ s, respectively, and each of these windows was also taken as a 5-s interval centered on the corresponding time. This choice is consistent with the typical geometry and speed of a 16-car high-speed train: with a train length of approximately 400 m and a speed of approximately 80 m/s, the train passage lasts about 5 s. Therefore, the central window approximately covers the complete train-passage interval, whereas the pre-arrival and post-departure windows correspond to times when the train is at least about one train length away from the observation point. The spectrum of the passage window was used to estimate the train frequency, and the spectra of the pre-arrival and post-departure windows were used to estimate the bridge frequency. The corresponding spectral results are shown in Figure 7b.

**Figure 8:**
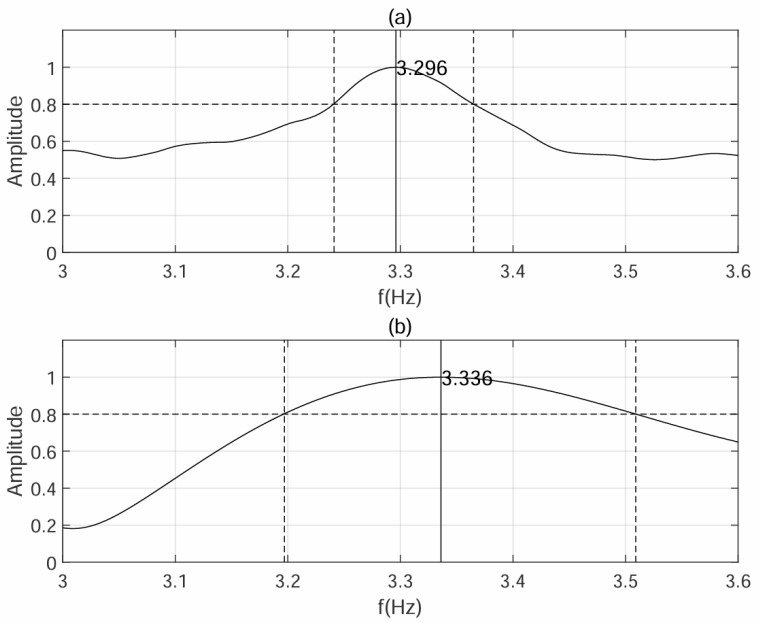
Measured results of the “train frequency” from real data using (**a**) the frequency-domain correlation method and (**b**) the cepstrum method. The horizontal dashed line marks the 80% peak-amplitude level, and the vertical dashed lines mark the corresponding peak-width interval. These markers are used as a consistent visual indicator of relative measurement uncertainty under the same threshold; the wider interval in (**b**) shows that the cepstral peak is less sharply localized than the correlation peak.

Using the frequency spectrum from the second row of Figure 7b, corresponding to the time window during train passage, the train frequency of this particular high-speed train was estimated using both the frequency-domain correlation method and the cepstral analysis. The results were 3.296 Hz and 3.336 Hz, respectively, as shown in Figure 8.

For this single-train example, the frequency-domain correlation curve gives a narrower peak-width interval than the cepstral curve, indicating higher local frequency resolution under the adopted reference spectrum and search settings. This does not imply that frequency-domain correlation is universally superior. Its advantage depends on the suitability of the assumed reference spectrum and the chosen search range, whereas the cepstral method has the practical advantage of estimating the spectral spacing directly without exhaustive template searching. The larger cepstral peak width observed here mainly reflects the finite spectral bandwidth and the weaker regularity of the field spectral lines. Thus, the two estimates should be viewed as complementary measurements: their difference provides an indication of method-dependent uncertainty, while their agreement within a narrow frequency range supports the robustness of the train-frequency estimate.

For the estimation of the bridge frequency, although the theoretical value is approximately 2.5 Hz, the energy at this fundamental frequency is often weak due to frequency modulation of the high-speed train seismic source, while the second-harmonic energy near 5 Hz appears most prominent, followed by the fourth-harmonic energy near 10 Hz [27].

For peak detection, the search was constrained to the expected harmonic bands instead of the full spectrum. Around the second harmonic, the amplitude spectra of the pre-arrival and post-departure windows were evaluated from 4.5 to 5.5 Hz with a frequency interval of 0.001 Hz, and the maximum amplitude in this interval was taken as the peak frequency. Around the fourth harmonic, the same procedure was applied from 9.0 to 11.0 Hz. The background spectra in Figure 6c,d were used to confirm that these harmonic bands were above the ambient noise level; no manually tuned global amplitude threshold was introduced. In the multi-trace analysis, peak estimates were not subjectively removed: values falling away from the stable cluster were retained and displayed as outliers, and the reported standard deviations therefore include their contribution. This is the reason that the fourth-harmonic estimates, which show fewer outliers, are treated as more reliable in the following analysis.

**Figure 9:**
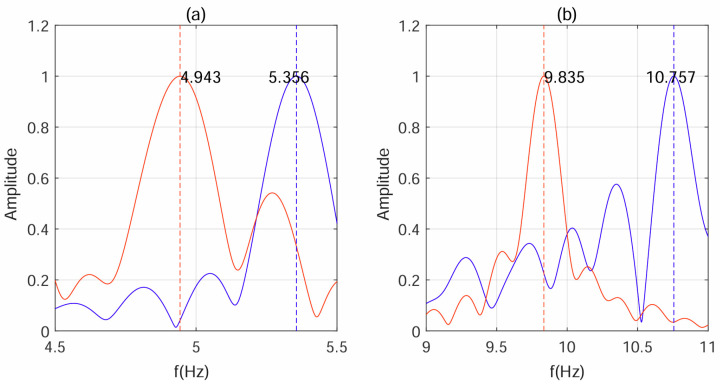
Measurement of bridge frequency from real data. (**a**) Spectral peak near the second harmonic (~5 Hz); (**b**) Spectral peak near the fourth harmonic (~10 Hz). Blue and red curves correspond to the normalized spectrum before train arrival and after train departure, respectively.

Figure 9 presents the normalized spectral segments around these harmonic frequencies, along with the annotated peak values. The results clearly exhibit a near 2:1 ratio between the detected frequencies, indicating consistency and reliability. Using these two frequency measurements and applying Equation (13), the estimated bridge frequencies are 2.571 Hz and 2.568 Hz, respectively. The small discrepancy (<0.005 Hz) confirms the robustness of this approach.

**Figure 10:**
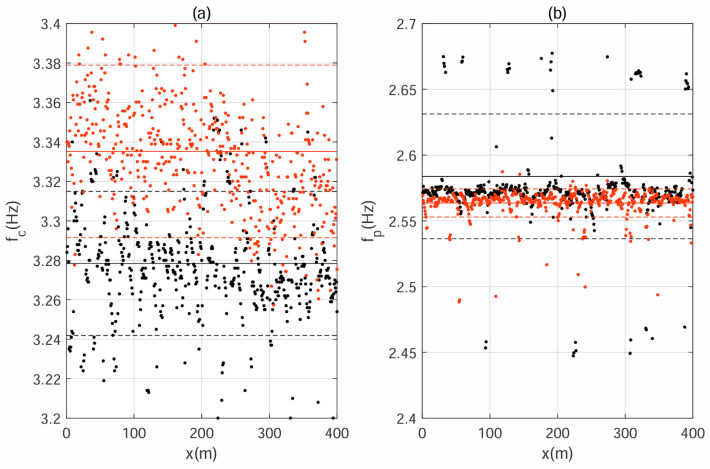
Train and bridge frequency measurements across 500 DAS traces. The solid lines denote the mean values, and the dashed lines denote the mean plus or minus one standard deviation, which are used here as trace-level uncertainty bands rather than population confidence intervals. (**a**) Distribution of train-frequency estimates: black dots represent results from frequency-domain correlation, and red dots represent results from cepstral analysis. (**b**) Distribution of bridge-frequency estimates: black dots correspond to measurements near 5 Hz (second harmonic), and red dots correspond to measurements near 10 Hz (fourth harmonic). Points far from the dashed uncertainty bands are retained as outliers and are discussed in the text.

The weak fundamental component should therefore be regarded as a limitation of direct fundamental-frequency picking rather than as an inherent instability of the proposed bridge-frequency estimator. In the HSR-DAS records, low-frequency components can be suppressed by source modulation, propagation attenuation, instrument response, cable–ground coupling, and interference from other wavefield components. A harmonic component with stronger spectral energy and higher signal-to-noise ratio can provide a more stable peak than the fundamental component. Because the approaching and receding peaks are paired within the same harmonic order, the measured Doppler-shifted harmonic frequencies can be converted back to the intrinsic bridge frequency after accounting for that harmonic order. Thus, the harmonic selection is signal-to-noise-ratio driven rather than arbitrary. The close agreement between the second- and fourth-harmonic estimates in Figure 9, together with the smaller trace-to-trace scatter of the fourth-harmonic picks in Figure 10b, indicates that using the clearest harmonic band improves the practical stability of bridge-frequency measurement under the present field conditions.

### 5.3. Multi-Trace Measurement Results

For the high-speed train passage analyzed in the previous subsection, both the train frequency and bridge frequency were estimated across all 500 observation traces using the previously described methods. The results are shown in Figure 10.

Figure 10a presents the train frequency measurements. A noticeable systematic bias is observed in the cepstral analysis results, which tend to be slightly higher than those obtained from the frequency-domain correlation method. The mean value from the frequency-domain correlation analysis is 3.278, with a standard deviation of 0.036. The mean value from the cepstrum method is 3.335, with a standard deviation of 0.043.

The mean difference between the two methods is approximately 0.057 Hz. Despite this bias, both methods yield a comparable standard deviation of about 0.04 Hz across the observation points, indicating similar consistency.

The error behavior in Figure 10a can therefore be separated into two parts: a method-dependent systematic difference, represented by the mean offset of approximately 0.057 Hz between the two estimators, and trace-dependent random scatter, represented by the standard deviations across the 500 DAS traces. The similar standard deviations of the two methods indicate that neither method is uniformly superior in all field traces. Instead, the frequency-domain correlation result is sharper when the source model is well matched, while the cepstral result is less dependent on assumed train parameters but more sensitive to the clarity and bandwidth of the equidistant spectral lines. In practical applications, the two methods can be used together: consistent estimates increase confidence in the measured train frequency, whereas systematic differences between them indicate model mismatch, spectral leakage, or local DAS coupling effects.

Figure 10b shows the bridge frequency measurements based on harmonic peaks. The estimates near 5 Hz (second harmonic) exhibit several outliers with large deviations from the mean, leading to a relatively high standard deviation. These outliers were not removed; they are retained to show the trace-local instability of the weaker second-harmonic picks. Because the deviations occur only at limited observation traces rather than coherently across the whole array, they are more likely related to local DAS coupling or cable–ground contact variations than to a change in the intrinsic bridge frequency. In contrast, the measurements near 10 Hz (fourth harmonic) are much more stable, with a standard deviation of only ~0.01 Hz, making them more reliable for back-calculating the bridge frequency.

**Figure 11:**
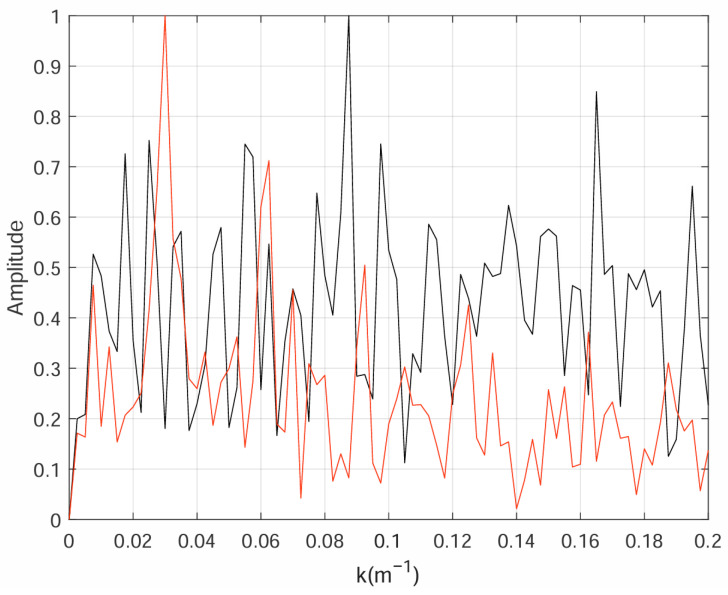
Spatial periodicity of train (black) and bridge (red) frequency measurements.

From the multi-point measurement results—particularly the black dots in Figure 10 representing the frequency-domain correlation estimates—an apparent spatial periodicity can be observed. To further examine this behavior, we performed spatial Fourier transforms on the measured train frequency and bridge frequency values. The results are presented in Figure 11.

As shown, the spatial spectrum of the train frequency exhibits a prominent peak around 0.09 m^−1^, corresponding to a spatial wavelength of approximately 11 m. In contrast, the bridge frequency spectrum shows a strong peak near 0.03 m^−1^, corresponding to a wavelength of about 32 m, which closely matches the actual bridge pier spacing. This observation suggests that the spatial variation in the bridge frequency is likely influenced by the geometric relationship between the sensing points and the positions of the bridge piers.

### 5.4. Multi-Train Measurement Results

**Figure 12:**
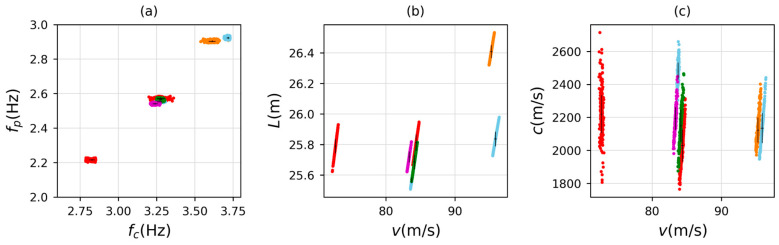
Measurement results for multiple trains: (**a**) train frequency $f_{c}$ and bridge frequency $f_{P}$; (**b**) carriage length and train speed; (**c**) wave velocity. Different colors represent different trains. The clusters in (**b**) are interpreted as operating-speed and carriage-length groups; they are not used as independently verified train-set labels. Formal uncertainty statistics for the multi-train results are given in the text. Crosses indicate the mean values, with their horizontal and vertical lengths representing the standard deviations of the variables corresponding to the horizontal and vertical axes, respectively.

From the one-hour DAS recording shown in Figure 6, we selected 8 individual trains with no interfering signals at least 100 s before and after each event. For each train, both train frequency and bridge frequency were measured across all 500 traces. The results are shown in Figure 12a.

The eight events were selected using the same signal-quality criterion: the 100 s intervals before and after each train event had to be free from interfering train passages or other strong non-train disturbances so that the weak pre-arrival and post-departure spectra required for bridge-frequency estimation could be measured reproducibly. No event was selected or rejected according to its estimated train frequency, bridge frequency, speed, or carriage length. Therefore, this subset is intended for transparent validation of the measurement workflow under interpretable field conditions rather than for statistical sampling of all train operations during the month-long deployment. The repeated frequency clusters across the selected trains and 500 DAS traces indicate that the conclusions are not driven by a single favorable event.

The measured frequencies cluster into three distinct groups, and a proportional relationship between the train and bridge frequencies is evident. This reflects their mutual dependence on train speed.

A comparison of the measurement variability reveals that the bridge frequency is more stable, with a smaller standard deviation than the train frequency. This suggests that bridge frequency provides a more reliable basis for estimating other physical parameters such as carriage length and train speed. These estimates are shown in Figure 12b.

To move beyond visual comparison, we further quantified the uncertainty of the multi-train measurements using the eight trains and 500 DAS traces per train shown in Figure 12. At the train level, the standard deviation of train frequency was significantly larger than that of bridge frequency (mean SD: 0.0640 Hz for $f_{c}$ and 0.0129 Hz for $f_{P}$; median SD ratio $f_{c}/f_{P}$ = 4.36, bootstrap 95% CI: 2.00–8.57). A paired *t*-test on log-transformed standard deviations confirmed this difference (t(7) = 5.27, *p* = 0.0012), and the non-parametric Wilcoxon signed-rank test gave the same conclusion (W = 36.0, *p* = 0.0039). The coefficient of variation also remained significantly smaller for bridge frequency (median CV ratio $f_{c}/f_{P}$ = 3.40; paired *t*-test, *p* = 0.0267; Wilcoxon test, *p* = 0.0078). At the trace level, using all 4000 paired trace measurements, the median normalized absolute deviation was 0.459% for $f_{c}$ and 0.107% for $f_{P}$, and the paired Wilcoxon test again indicated a significant reduction in uncertainty for bridge frequency (*p* < 0.0001). We also checked the two Doppler-shifted bridge-frequency components and the event-level signal-to-noise ratio. The combined bridge-frequency estimator was more stable than the post-departure shifted component (Wilcoxon test, *p* = 0.0195), while the pre-arrival component was already comparable to the combined estimator (*p* = 0.7695). Across the event-level SNR range of 38.6–50.6 dB, the bridge-frequency advantage persisted in both lower- and higher-SNR subsets, and the stability ratio showed no systematic dependence on SNR (Spearman rho = 0.02, *p* = 0.9554). These formal uncertainty tests support the interpretation that bridge frequency is a more stable frequency eigenquantity for subsequent parameter estimation.

The carriage lengths cluster around approximately 25.75 m, 25.90 m, and 26.35 m, and the corresponding speeds also form several operating groups. Because independent train-set identification information was not available for each passage, these clusters should be interpreted as physically reasonable operating-speed and carriage-length groups rather than definitive train-type assignments. Their correspondence with typical Chinese HSR speed classes and carriage lengths provides a plausible interpretation, but the main conclusion drawn from Figure 12 is the repeatable clustering of the frequency-derived parameters and the lower uncertainty of bridge-frequency measurements, not exact train-model classification.

Furthermore, we used the Doppler-shifted frequencies obtained during bridge-frequency measurement to estimate an apparent wave propagation velocity. The results are shown in Figure 12c. The estimated velocities are relatively consistent across different trains and cluster around 2200 m/s, providing an internal consistency check for the Doppler-based frequency measurements. We emphasize that an independent true P-wave velocity was not available for the deployed railway segment. Such a true value is difficult to obtain near an operating HSR viaduct using conventional controlled-source surveys, whereas passing high-speed trains provide one of the few repeatable and energetic sources available along the line. Therefore, the velocity result is interpreted here as a preliminary apparent velocity estimate rather than a fully calibrated site-velocity model. Because the reported S-wave and surface-wave velocities in loose sediments of the North China Plain are on the order of several hundred meters per second [13], the measured apparent velocities are more consistent with a P-wave-related component or elastic-wave propagation along the bridge structure.

However, the standard deviation of wave velocity measurements across different observation points is approximately 200 m/s, indicating notable trace-to-trace variability. This scatter is likely related to the presence of multiple seismic phases in the recorded wavefield, which may interfere with frequency peak identification. Further analysis and independent calibration are needed to improve precision.

Because the shallow subsurface in and around the study area consists of loose sediments, the S-wave and surface-wave velocities are generally much lower than the measured apparent velocity and are typically on the order of several hundred meters per second [13]. Therefore, the observed apparent velocity of approximately 2200 m/s is more consistent with a P-wave-related component or a bridge-guided elastic-wave component than with the dominant surface-wave component. This interpretation is not a substitute for independent velocity calibration, but it is physically plausible under the present observation geometry. Given that the actual seismic wavefield contains multiple phases, including P-waves, S-waves, and surface waves, the possible prominence of the P-wave-related Doppler-effect peak can be explained by three factors:

(a) Polarization effect: In our analysis, the selected records correspond to situations where the train is located on either side of the observation point, meaning that seismic energy propagates laterally from the source to the receiver. Because the fiber-optic cable is installed parallel to the railway line, the DAS system is more sensitive to horizontally propagating P-waves, enhancing their contribution to the recorded signal.
(b) Attenuation effect: In loose near-surface sediments, S-waves and surface waves attenuate more rapidly than P-waves. As a result, P-waves can propagate over longer distances, making them more likely to dominate the recorded wavefield at the observation point.
(c) Surface waves generally exhibit lower frequencies (approximately 3 Hz in actual high-speed railway observations). In contrast, the Doppler-effect calculations in this study are conducted at 5 Hz and 10 Hz, which exceed the observed frequency range of surface waves and are therefore more likely associated with body waves.

## 6. Conclusions

This study investigates the characteristic frequency components in high-speed railway (HSR)-induced seismic wavefields and draws the following main conclusions:

Building on previous theoretical work on HSR seismic sources, this study systematizes the measurement of two frequency eigenquantities rather than claiming that all underlying spectral phenomena are new. The novelty of this work lies in the integrated measurement framework: frequency-domain correlation and cepstral analysis are compared for train-frequency estimation, paired Doppler-shifted components are combined to obtain a velocity-independent bridge-frequency estimator, and the resulting measurements are evaluated with dense DAS traces across single-train, multi-trace, and multi-train settings. The proposed workflow is validated through synthetic tests and real DAS observations, with measurement uncertainty evaluated across dense sensing traces and multiple trains.

For single-train, multi-trace measurements from real DAS recordings, the standard deviation of train-frequency estimates is approximately 0.03 Hz, while that of bridge-frequency estimates is around 0.01 Hz, indicating that bridge frequency offers higher measurement stability and accuracy. Moreover, the results exhibit spatial periodicity, likely related to the distribution of bridge piers, suggesting that the relative geometry between sensors and bridge structures should be considered in future analyses.

In the multi-train analysis, train-frequency and bridge-frequency estimates show a strong positive correlation, consistent with theoretical predictions. The estimated train speeds and carriage lengths based on bridge frequency are consistent with typical HSR operating speeds and carriage-length ranges in China. Compared with conventional train-frequency-based speed estimation, the bridge frequency provides more stable parameter estimates in the analyzed DAS records and offers a possible route for inferring wave velocity.

The results presented in this study provide a theoretical and methodological basis for quantitative source-frequency characterization in DAS-based HSR vibration studies. Extending this workflow to operational safety monitoring, train-type recognition, or structural health diagnostics will require further validation using independently labeled train information and structural-condition data.

## Data Availability

The data presented in this study are available from the corresponding author upon reasonable request.

## References

[1] Giunta M. (2023). Trends and challenges in railway sustainability: The state of the art regarding measures, strategies, and assessment tools. Sustainability.

[2] Hacıefendioğlu K., Mostofi F., Aslan T., Toğan V. (2026). A Dual-Domain Deep Convolutional Variational Autoencoder Framework for Unsupervised Structural Health Monitoring Using Vision-Based Vibration Analysis. J. Comput. Civ. Eng..

[3] Bao T., Ning J., Zhang X. (2019). Extraction of characteristics of wavefield under viaduct produced by high-speed rail. Beijing Da Xue Xue Bao.

[4] Hu G., Zhang G., Sun S., Li Y., Yang J., Duan J., He H., Zhan Y. (2019). FWI of high-speed-train seismic first arrival signal. Chin. J. Geophys..

[5] Jiang Y., Bao T., Ning J., Zhang X. (2019). Spectral characteristics of high-speed rail seismic signal under viaduct. Beijing Da Xue Xue Bao.

[6] Wen J., Ning J., Zhang X. (2019). Theoretical analysis on the characteristics of seismic wave field produced by high-speed train. Beijing Da Xue Xue Bao.

[7] Fang G., Li Y.E., Zhao Y., Martin E.R. (2020). Urban near-surface seismic monitoring using distributed acoustic sensing. Geophys. Res. Lett..

[8] Song Z., Zeng X., Thurber C.H. (2021). Surface-wave dispersion spectrum inversion method applied to Love and Rayleigh waves recorded by distributed acoustic sensing. Geophysics.

[9] Wang B., Zeng X., Song Z., Li X., Yang J. (2021). Seismic observation and subsurface imaging using an urban telecommunication optic-fiber cable. Chin. Sci. Bull..

[10] Liu H.Y., Ma J.H., Yan W.F., Liu W.S., Zhang X., Li C.C. (2018). Traffic Flow Detection Using Distributed Fiber Optic Acoustic Sensing. IEEE Access.

[11] Liu H.Y., Ma J.H., Xu T.W., Yan W.F., Ma L.L., Zhang X. (2020). Vehicle Detection and Classification Using Distributed Fiber Optic Acoustic Sensing. IEEE Trans. Veh. Technol..

[12] Cai Y.P., Yan W.F., Liu H.Y., Sun Y.T., Zhou X.L. (2020). Security Monitoring of Smart Campus Using Distributed Fiber Optic Acoustic Sensing. AOPC 2020: Optical Information and Network.

[13] Shao J., Wang Y., Chen L. (2022). Near-surface characterization using high-speed train seismic data recorded by a distributed acoustic sensing array. IEEE Trans. Geosci. Remote Sens..

[14] An Y., Ma J., Xu T., Cai Y., Liu H., Sun Y., Yan W. (2023). Traffic Vibration Signal Analysis of DAS Fiber Optic Cables with Different Coupling Based on an Improved Wavelet Thresholding Method. Sensors.

[15] Fredriksen S.L.B., Mai T.T., Growe K., Eidsvik J. (2024). Tracking and classifying objects with DAS data along railway. arXiv.

[16] Liu J., Li H., Yuan S., Noh H.Y., Biondi B. (2024). Characterizing Vehicle-Induced Distributed Acoustic Sensing Signals for Accurate Urban Near-Surface Imaging. arXiv.

[17] Molenaar M.M., Hill D.J., Webster P., Fidan E., Birch B. (2012). First Downhole Application of Distributed Acoustic Sensing for Hydraulic-Fracturing Monitoring and Diagnostics. SPE Drill. Complet..

[18] Parker T., Shatalin S., Farhadiroushan M. (2014). Distributed Acoustic Sensing–a new tool for seismic applications. First Break.

[19] Dou S., Lindsey N., Wagner A.M., Daley T.M., Freifeld B., Robertson M., Peterson J., Ulrich C., Martin E.R., Ajo-Franklin J.B. (2017). Distributed acoustic sensing for seismic monitoring of the near surface: A traffic-noise interferometry case study. Sci. Rep..

[20] Rodríguez Tribaldos V., Ajo-Franklin J.B. (2021). Aquifer monitoring using ambient seismic noise recorded with distributed acoustic sensing (DAS) deployed on dark fiber. J. Geophys. Res. Solid Earth.

[21] Takemiya H., Bian X.C. (2007). Shinkansen high-speed train induced ground vibrations in view of viaduct–ground interaction. Soil Dyn. Earthq. Eng..

[22] Chen Q., Li L., Li G., Chen L., Peng W.-T., Tang Y., Chen Y., Wang F.-Y. (2004). Seismic features of vibration induced by train. Acta Seismol. Sin..

[23] Wang X., Wang B., Chen W., Li J. (2019). Using the data from one receiver to estimate running velocity of high-speed train. Beijing Da Xue Xue Bao.

[24] Lavoué F., Coutant O., Boué P., Pinzon-Rincon L., Brenguier F., Brossier R., Dales P., Rezaeifar M., Bean C.J. (2021). Understanding seismic waves generated by train traffic via modeling: Implications for seismic imaging and monitoring. Seismol. Res. Lett..

[25] Fuchs F., Bokelmann G., AlpArray Working Group (2018). Equidistant spectral lines in train vibrations. Seismol. Res. Lett..

[26] Xu S., Guo J., Li P., Jiang M.W. (2017). Observation and analysis of ground vibrations caused by the Beijing-Tianjin high-speed train running. Prog. Geophys..

[27] Jiang Y., Ning J., Wen J., Shi Y. (2022). Doppler effect in high-speed rail seismic wavefield and its application. Sci. China Earth Sci..

[28] Quiros D.A., Brown L.D., Kim D. (2016). Seismic interferometry of railroad induced ground motions: Body and surface wave imaging. Geophys. J. Int..

[29] Liu Y., Yue Y., Li Y., Luo Y. (2021). On the retrievability of seismic waves from high-speed-train-induced vibrations using seismic interferometry. IEEE Geosci. Remote Sens. Lett..

[30] Wen J., Shi Y., Ning J. (2021). Measurement of high-speed rail surface-wave phase-velocity dispersion. Chin. J. Geophys..

[31] Shi Y., Wen J., Ning J. (2022). Theoretical analysis of high-speed rail seismic imaging. Sci. China Earth Sci..

[32] Zhang H., Wang B., Ning J., Li Y. (2019). Interferometry imaging using high-speed-train induced seismic waves. Chin. J. Geophys..

[33] Mi B., Xia J., Xu Y., You B., Chen Y. (2023). Retrieval of surface waves from high-speed-train-induced vibrations using seismic interferometry. Geophysics.

